# Women's Experiences of Care and Support Following Postpartum Psychosis: A Meta‐Ethnography

**DOI:** 10.1111/jan.70195

**Published:** 2025-09-03

**Authors:** Vimbai Carr, Gill Thomson, Victoria Moran, Gill Strachan

**Affiliations:** ^1^ University of Central Lancashire Preston UK

**Keywords:** childbearing, meta‐ethnography, midwifery, mother and baby unit, nurse, nursing, perinatal mental health, postpartum psychosis, psychology, qualitative approaches

## Abstract

**Background:**

Postpartum psychosis is a psychiatric emergency that occurs following childbirth. Women are often cared for in general psychiatric units or in psychiatric Mother and Baby units. Postpartum psychosis is associated with a significant risk of relapse. There is a need to explore how women perceive care to understand what works well or needs further improvement.

**Aims:**

This review aimed to explore women's experiences of care and support for postpartum psychosis.

**Design:**

A systematic review using meta‐ethnographic methods was conducted.

**Data Sources:**

Comprehensive searches were conducted between 4 March 2024 and 4 March 2025 on five databases (CINAHL, EMBASE, MEDLINE, PsycINFO and Web of Science). Backward and forward chain searching was also undertaken.

**Review Methods:**

Critical appraisal was conducted following screening. Reciprocal and refutational translation were used to form the synthesis, and a line of argument was developed. The eMERGe reporting guidelines were used.

**Results:**

Fifteen studies were included within this synthesis. All the studies were conducted in high income countries and included 235 women. Three main themes were developed. ‘Navigating the unknown’ explored women's perceptions of postpartum psychosis as a less well‐known condition, and their informational needs. ‘The double‐edged sword of care’ found that there were helpful elements of formal mental health care, but that accessing care was sometimes traumatic, stigmatising and conflicting to women's identities. ‘Seeking consolation and recovery’ explored women's need for psychological support and experiences of peer support.

**Conclusion:**

The findings of this review highlighted women's needs in respect to informational support, medication support, psychological support and in‐patient care settings. Mother and baby units were strongly preferred by women.

**Impact:**

The findings highlighted a need for specialised care for postpartum psychosis.

**Patient or Public Contribution:**

There were no patient or public contributions.

**Trial Registration:**

Prospero (CRD42024515712)

**Summary**

- Problem or issue
  ○ Women with postpartum psychosis are associated with an increased risk of suicide or harm to the infant, and have significant rates of relapse and readmission.
  ○ There is a need for further research into the care and support needs of women with postpartum psychosis to understand how care may be improved to enhance outcomes.
- What is already known.
  ○ Women with postpartum psychosis are likely to experience variation in care experiences due to uncertainties in diagnosis, and availability and variations in care pathways.
- What this paper adds.
  ○ This qualitative synthesis highlights women's experiences of care across different care settings.
  ○ This paper highlights that women with postpartum psychosis wanted care that did not just treat their psychiatric symptoms but considered their needs as mothers.
  ○ The care in Mother and Baby units was preferred and perceived as more appropriate for mothers than care in general psychiatric units.
  ○ This review also highlights women's needs for information, further education of their wider support network and healthcare professionals, peer support and psychological support.

## 1 Introduction

Postpartum psychosis (PP) is a rare psychiatric emergency that occurs in the early postnatal period (Raza and Raza 2024). PP is estimated to affect between 1 and 3 women per 1000 births (Kalra et al. 2022; Vander kruik et al. 2017) or up to 1 in 5 women with bipolar disorder (Wesseloo et al. 2016). Women with PP may experience symptoms such as mood lability, insomnia, visual or auditory hallucinations, delusions and paranoia (Raza and Raza 2024). Women's memory and decision‐making may be severely affected during this time (Spinelli 2021) and they may experience suicidal thoughts (Brockington 2017; Friedman et al. 2023). Delusions in PP are often centred around the infant and may result in maternal anxiety, infant harm, or affect mother‐infant interactions (Chandra et al. 2006). Infanticide occurs in 1%–4.5% of cases of PP (Friedman et al. 2023). Evidence suggests that problems in screening/identification of PP, missed opportunities for intervention and treatment and problems in accessing care are common factors in mothers who committed infanticide (Alford et al. 2024). There is a lack of consensus about the number of symptoms required for a diagnosis of PP, duration of symptoms, the associated risk (Sharma et al. 2022) and the Diagnostic and Statistical Manual of mental disorders (DSM) at present does not class PP as a separate and distinct diagnosis. This may contribute to a lack of understanding about PP and the associated care and support needs of women.

Current care for women with PP and other perinatal mental illnesses varies in terms of care settings, interventions, admission length and follow‐up after discharge within and outside of the UK (Gillham and Wittkowski 2015; Hauge et al. 2024). In the UK, mothers can access a range of services including community services and inpatient services—with some services being specialised to perinatal needs, and some services being broader. Most women are cared for in general psychiatric units where they are separated from their infant. Alternatively, mothers can be admitted into psychiatric Mother and Baby Units (MBUs) which allow mothers to be admitted with their baby. Access to MBUs that support co‐admission with the infant is limited within the UK and internationally. Women with PP often also require further support following discharge (Friedman et al. 2023; Kobylski et al. 2024). Women who have experienced PP are associated with a high relapse rate—it is estimated that up to one in two women will relapse in a subsequent pregnancy following PP (Blackmore et al. 2013; Wesseloo et al. 2016; Robertson et al. 2005). There is limited understanding of how care and support can be improved to support women with PP and prevent relapse and readmission (Forde et al. 2020; Howard et al. 2022; Hauge et al. 2024). Limited studies have explored mothers' experiences of care across UK perinatal services (Roxburgh et al. 2023; Griffiths et al. 2019; Howard et al. 2022) and to our knowledge there is no review focused on the care of women with PP as a primary focus. Previous qualitative research has provided helpful insights into mothers' lived experiences of postpartum psychosis (Forde et al. 2020; Wicks et al. 2019), factors involved in recovery (Forde et al. 2020) and perspectives of fathers/partners (Lyons et al. 2023). A recent systematic review exploring the research conducted in MBUs in relation to patients, their families and staff found gaps in knowledge regarding long‐term outcomes of women, and what services were available or accessed by women following discharge (Adhikary et al. 2024). This review, however, included women with different diagnoses, not solely PP. Therefore, this systematic review using meta‐ethnographic methods was undertaken to identify and synthesise the current qualitative evidence base on women's experiences of care and support following PP from treatment during the acute episode to post‐discharge.

## 2 Methods

### 2.1 Review Question

The following research question was developed to guide this review: ‘What are mothers' experiences of care and support following postpartum psychosis?’

### 2.2 Review Methodology

Meta‐ethnography (Noblit and Hare 1988) is a method used to develop new interpretations or theories from primary qualitative studies (Walsh and Downe 2005): this approach was felt to be appropriate for the current review as little is known about the care and support that may be helpful or unhelpful for women with PP. Meta‐ethnography aims to find ‘meaning in context’ (Noblit and Hare 1988); considering the context of primary studies within interpretations was important in this review as women received care across different settings including community, hospital settings, MBUs and general psychiatric units.

A systematic approach was used to search, identify and select qualitative‐based studies, with the aim to reduce bias and thus provide more confidence in the robustness of the findings. The eMERGe reporting guidelines for meta‐ethnographies were used to guide the reporting of this review (France et al. 2019). The review protocol was registered on PROSPERO (CRD42024515712) (Carr et al. 2024).

### 2.3 Search Strategy

The search strategy was developed by the review team and in consultation with an information specialist. A PEO framework (population, exposure, outcomes) was used to frame the research question, to develop search terms for the search strategy (including truncation) and inclusion and exclusion criteria (see Table 1). The decision to exclude outcome terms (from the PEO framework) from the search terms was made to enable a wider search following trial pilot searches.

**TABLE 1:** Search criteria and inclusion and exclusion criteria.

	Inclusion criteria	Exclusion criteria	Search terms
Population	Women/mothers/birthing people who have experienced postpartum psychosis	Described multiple perinatal disorders where findings relating to postpartum psychosis cannot be distinguished Staff, partner, other perspectives	Wom*n mother* mum* mom* birthing person* birthing people
Exposure	Studies exploring mothers' experiences of care and support following postpartum psychosis	Exploring experiences not related to care and support; or of perinatal mental illnesses/disorders	Psychos*s or Psychotic Disorder or Psychotic illness AND post‐natal or post‐partum or postnatal or puerperal or puerperium or peripartum or peri‐partum or pregnancy or childbirth or birth or perinatal or peri‐natal
Outcome	Views, experiences, opinions, perceptions, feelings, acceptability, engagement, perspectives	Quantitative outcomes	
Language	Studies published in any language (whereby translation software can be used to translate appropriately)	Articles unable to be effectively translated	
Study type	Empirical research using primary qualitative methods	Secondary research Quantitative‐based studies	
Publication type	Peer reviewed journal articles	Grey literature, case studies, opinion pieces, commentaries, editorials	

Qualitative studies whereby women with PP reported experiences of care and support were included. Qualitative studies where mothers did not report experiences of care or support for PP were excluded. Only primary peer‐reviewed qualitative studies were included. Mixed‐method studies were eligible for inclusion provided that qualitative data could be extracted. No restriction was placed on the year of publication to enable a wider search.

Studies were excluded if they explored women's experiences of multiple perinatal disorders whereby experiences of women with PP could not be isolated, or if they explored multiple person perspectives whereby women's perspectives could not be independently extracted. Secondary research and quantitative research were excluded. Articles that could not be effectively translated were excluded. Full inclusion and exclusion criteria are included in Table 1.

### 2.4 Search Process and Study Selection

A comprehensive search was conducted between 4 March 2024 and 04 March 2025 on databases: CINAHL, EMBASE, MEDLINE, PsycINFO and Web of Science. Forward and backward searching was also used. Identified articles were downloaded to Endnote. All articles were then uploaded to Rayyan (an online software programme https://www.rayyan.ai). Duplicates were removed using Endnote, Rayyan and manually. Articles were initially screened by title and abstract. Double‐blind screening occurred on 20% of articles. Three conflicts were found and were discussed amongst reviewers until there was consensus (inter‐rater reliability (99.43%)). As there was good inter‐rater reliability amongst reviewers, one reviewer completed the screening on the remaining articles. All the full‐text articles were double screened. The search process was conducted following PRISMA guidelines, and a PRISMA diagram was used to present the search results.

### 2.5 Quality Appraisal

Following screening and selection of studies, quality appraisal was carried out to assess the studies' methodological quality and risk of bias. While qualitative systematic reviews generally include some form of quality assessment, there is a lack of consensus on what is the best tool to use. The appraisal tool developed by Downe et al. (2009) was chosen due to its inclusion of questions about the research context and consideration of rigour. The appraisal tool has been utilised in other qualitative evidence syntheses to assess study quality (Thomson et al. 2019; Kuforiji et al. 2023; Downe et al. 2018). The tool includes questions on different aspects of quality, and each question can be answered as yes, no, or unclear. Following assessment of each qualitative aspect, each study was scored in accordance with the tool as: A (no/few flaws), B (some flaws‐ unlikely to affect quality), C (some flaws that may affect quality) or D (significant flaws that are likely to affect quality). Quality assessment was carried out by reviewers independently, then discussed until consensus was reached. The decision was made not to exclude any studies on quality, as some studies may be conceptually rich but have methodological limitations (Toye et al. 2014), and that any methodological issues would be highlighted when discussing the findings. Characteristics of included studies (including study type, methods, participants and other contextual information–e.g. setting of care) were recorded in Table 3. All papers were uploaded to MAXQda qualitative software to organise and analyse data.

### 2.6 Synthesis Approach

The guidance for undertaking a meta‐ethnography emphasises how the analysis phases are relatively fluid and non‐linear. Articles were read multiple times through the review process. The order in which studies are analysed can affect the synthesis as former papers may strongly influence the development of latter ideas (Sattar et al. 2021). Therefore, papers were analysed with respect to quality appraisal scores, starting with the highest scoring papers (A grade) and finishing with the lowest scoring paper (D grade). Data analysis involved coding second order constructs interpretations, themes or metaphors used by the paper authors (Flemming et al. 2019) into third order interpretations (the review team interpretations involving organising the data into themes and sub‐themes) and identifying first order constructs participant quotations/primary data that could be used as supporting evidence and to enhance the credibility of the findings. The initial third‐order interpretations (comprising themes and sub‐themes and supporting first‐order constructs) were discussed within the review team and then modified further. Analysis involved looking at how themes related to each other across papers. Reciprocal translation was used to synthesise complementary themes across the different studies. Refutational translation was used to synthesise key themes that were different or contradictory across studies. These common or dissimilar findings were then drawn together into a new conceptualisation or interpretation referred to as line of argument synthesis.

### 2.7 Reflexivity

The review team included four members from multiple disciplines including midwifery, psychology, maternal and infant nutrition and psychiatry. This offered different perspectives on care and support and prevented biases from a purely clinical perspective. All authors were involved in data analysis and in agreeing the final interpretations.

## 3 Findings

**FIGURE 1:**
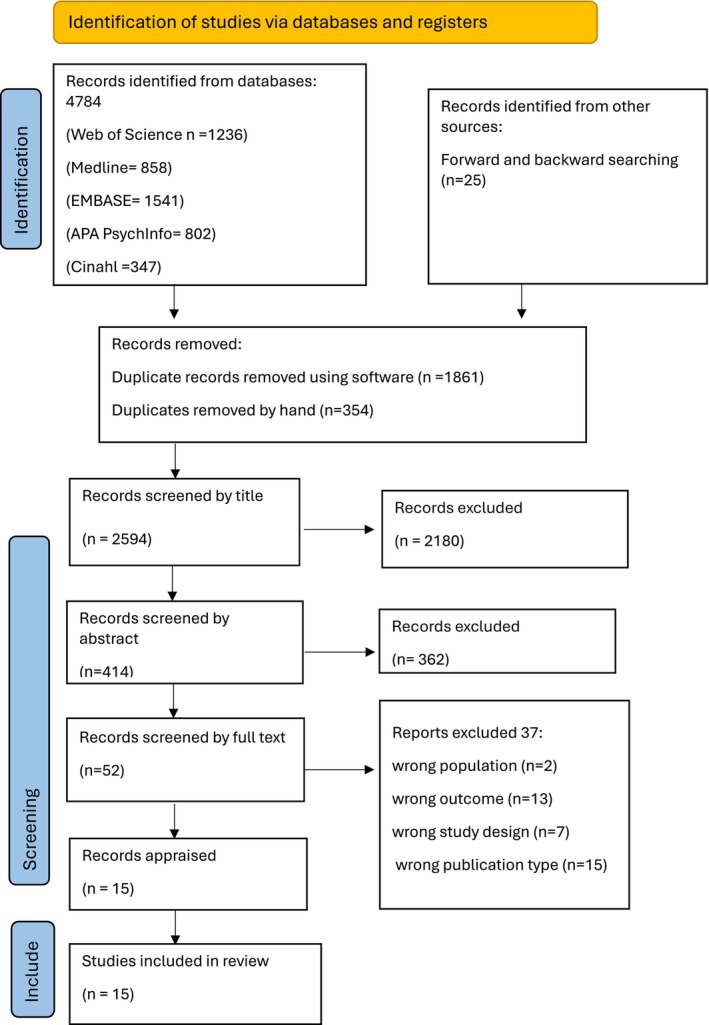
PRISMA diagram.

Database searches identified 4784 articles. A further 25 articles were identified by other sources including backward and forward searching. Following de‐duplication there were 2594 articles (see PRISMA diagram‐ Figure 1). Following screening, there were 52 publications for full text review and 15 were selected for inclusion. Reasons for exclusion included the wrong population (*n* = 2), wrong outcome (*n* = 13), wrong study design (*n* = 7) and wrong publication type (*n* = 15).

Studies were conducted in high‐income countries, i.e., in Sweden (*n* = 1), UK (*n* = 9), Australia (*n* = 1), USA/Canada (*n* = 3) and a latter study was carried out in Sweden but used online narrative data sorted from the internet (*n* = 1). There were 229 participants in total (majority of samples included 5–20 women). Participants were cared for in a variety of care settings including hospital/general psychiatric units (*n* = 160), MBUs (*n* = 32) and home/community settings (*n* = 9). Most women in the included studies were white, employed, married, heterosexual and/or had a partner. Time since onset of PP ranged from 3 months to 37 years. The majority of studies used interviews (*n* = 14), and one study used cross‐case analysis of online narratives (*n* = 1). Study characteristics and quality appraisal scores are presented in Table 2.

**TABLE 2:** Summary table of included study characteristics and quality appraisal scores.

Author, year	Country	Aim	Sample description	Care setting	Design & Methods	Key themes	QA score
Engqvist and Nilsson (2014)	Sweden	To explore women's experiences of PP	13 participants Time since onset of PP: ranged from 7 to 32 years for the women.	*n* = 12 hospital admission (*n* = 12), not hospitalised (*n* = 1)	Qualitative, Recorded interview, Inductive content analysis	(1)The recovery process, (2) the circumstances of support provided	B
Forde et al. (2019)	UK	To explore experiences, needs and preferences for psychological intervention from perspectives of women and their families	21 participants (13 women, 8 family members). Time since onset of PP: ranged from 3 months to 23 years. Characteristics of women (*n* = 13): university educated (*n* = 9), employed (*n* = 12), within a relationship (*n* = 11)	MBU care (*n* = 10), general psychiatric care (*n* = 7), A and E (no follow up) (*n* = 1), No treatment provider (*n* = 1)	Qualitative, semi‐structured interviews, Thematic Analysis	(1) Seeking safety and containment, (2) Recognising and responding to the psychological impact and (3) Planning for the future.	A—
Glover et al. (2014)	UK	To explore women's experiences of PP	7 participants. Time since onset of PP: up to 10 years Authors describe majority of women as employed.	All had hospital care	Qualitative, semi‐structured interviews, Inductive Thematic analysis	(1) The path to PP; (2) Unspeakable thoughts and unacceptable self; (3) ‘Snap out of it’; (4) Perceived causes	B+
Heron et al. (2012)	UK	To explore women's experiences of recovery and their beliefs about services needed for recovery to inform post discharge support	5 participants Time since onset of PP: 3–20 years Married (*n* = 5), employed (*n* = 5), British (*n* = 4), European (*n* = 1)	MBU (*n* = 1), general psychiatric unit (*n* = 3), private hospital (*n* = 1)	Qualitative design, Semi‐structured interviews, Grounded analytic induction approach	Unmet expectations, Ruminating and Rationalising, Social recovery, medical support, Information needs, family support, Giving recovery time	B
Jefferies et al. (2021)	Australia	To gain understanding of women's experiences of PP‐ recognition of PP, seeking support for PP, and contributing factors to PP	10 women Time since onset of PP: up to 10 years Within a relationship (*n* = 10)	Care settings not stated	Qualitative interpretive design, Semi‐structured interviews, Thematic analysis	Banks of the River: Family history or pre‐existing mental illness after a previous pregnancy. Muddy Waters: problems during pregnancy, childbirth or the early postnatal period, Gathering Momentum: Subtle changes in thoughts or behaviour, The Rapids: Symptoms of Psychosis, The Misty Pool: Recovery	B
Mcgrath et al. (2013)	UK	‘To develop a theoretical understanding of recovery from psychosis following childbirth’	12 mothers Time since onset of PP: below 1 year‐ 23 years (mean ~5 years) Employed (*n* = 9), Married/cohabiting (*n* = 10), White British (*n* = 12)	General psychiatric ward (*n* = 6), MBU (*n* = 2), maternity ward (*n* = 2), home (*n* = 1)	Grounded theory, Semi‐structured, Constant comparison method	Four themes including: (1) the process of recovery; (2) evolving an understanding; (3) strategies for recovery; and (4) sociocultural context.	A—
Roberts et al. (2018)	UK	To explore how the East‐ender's PP storyline was received by women who have recovered from PP	9 mothers Time since onset of PP: 3 years to 37 years White British (*n* = 9)	Care settings not stated	Semi‐structured interviews, Inductive analysis	Five themes: (1) public education, (2) stigma, (3) disclosure, (4) reassurance, (5) family relationships.	B
Robertson and Lyons (2003)	UK	To explore women's experiences of PP and living with the experience following illness	10 women Time since onset of PP: 2–10 years	Specialist mother and baby units and on general psychiatric wards, home	Semi structured interviews, Constant comparative analysis	Three main categories: puerperal psychosis as a separate form of mental illness, loss, and relationships and social rules	B—
Vanderkruik et al. (2024)	USA	To explore women's experiences of PP	130 women Time since onset of PP: up to 10 years White (*n* = 112), Black (*n* = 5), Asian (*n* = 4), Other (*n* = 9), bachelor's degree (*n* = 54), Postgraduate educated (*n* = 58), Employed (*n* = 103), Married (*n* = 119)	Hospital care	Interview‐ summarised and transcribed in real time (not recorded). Content analysis.	(1)Broad psychosocial experience, (2)mother‐baby dyad, (3) treatment and recovery experiences.	D
Roxburgh et al. (2023)	UK	To explore women's experiences of mental health services amongst women treated for PP	12 women Time since onset of PP: within first year White (*n* = 8), Asian (*n* = 2), Mixed (*n* = 1), Black (*n* = 1), Postgraduate education or above (*n* = 7), Living with partner (*n* = 11)	Mbu (*n* = 6), general psychiatric ward (*n* = 5), crisis house (*n* = 1), perinatal community mental health team (*n* = 1), Early Intervention in Psychosis community services (*n* = 2) (women accessed more than one service)	Qualitative, semi‐structured interviews, Thematic analysis	Six themes were identified (1) the influence of maternity care on women's mental health, (2) lack of knowledge, awareness and information on PP, (3) problems accessing care, (4) inpatient admissions: the need for a therapeutic environment, (5) value of consistency, cohesiveness and choice and (6) being mindful of the partner	B
Doucet et al. (2012)	USA & Canada (multi‐site)	To explore support needs and preferences of women with PP and their partners	9 women Time since onset of PP: 0–6 years (*n* = 8 within 2 years from PP onset). White (*n* = 7), Other (*n* = 2), University educated (*n* = 9), Married (*n* = 6), employed (*n* = 7)	General psychiatric unit (all)	Qualitative descriptive study, Semi structured interviews, Inductive thematic analysis	Main themes: (1)generic parenting needs, (2) serious mental illness needs.	B—
Engqvist et al. (2011)	Online narratives (countries not specified)	Gain insight in to women's experiences of PP	10 online narratives Time since onset of PP: unknown (Demographics unknown)	Unknown	Cross case analysis and content analysis	(1) unfulfilled dreams, (2)enveloped by darkness, (3)disabling symptoms, (4) feeling abandoned.	B
Posmontier and Fisher (2014)	USA	Understand the experience of PP in an Orthodox Jewish woman	1 woman	Hospital	Case Based narratology, Unstructured interview, Structured analysis	Segments/stanzas: (1)Birth and Shavuos; (2)the second day, (3)the symptoms begin; (3)the third day, Coming home; (4)the fourth day, Shabbos psychosis; (5) the aftermath, Making sense of the experience.	B
Wass et al. (2024)	UK	To understand impact of PP on the couple's relationship	14 participants (8 women) Time since onset of PP: 21 months to 9 years Characteristics of women within a relationship (*n* = 8).	MBUs (*n* = 10), general psychiatric units (*n* = 5), and community (*n* = 2), Community (*n* = 2) Participants cared for in more than one setting	Grounded theory, Semi‐structured interviews, Constant comparative analysis	(1)Our relationship before (2) Relationship test, (3) Picking up the pieces, (4) Discovering the new us	A—
Wyatt et al. (2015)	UK	To explore PP and women's relationships from perspectives of women and significant others	7 women Time since onset of PP: 5 months to 4 years Authors describe participants as predominantly white.	MBU (*n* = 3), Psychiatric (*n* = 4)	Interpretative phenomenological approach, Semi‐structured interviews, Interpretive phenomenological analysis	(1) ‘she wasn't herself’: (2) invalidation and isolation: (3) ‘the worst life can throw at us’: (4) a double‐edged sword: understanding relationships as negatively and positively influencing PP experience.	B

Regarding quality appraisal, three studies scored A grades and 11 studies were graded B. Strengths of most studies included the use of clear research aims and appropriate use of qualitative methodology to enhance rigour. Studies were downgraded due to lack of a theoretical framework/underpinning methodology (*n* = 6), lack of justification of sample size and recruitment strategy (*n* = 7), insufficient description of study context (*n* = 6), lack of reporting on reflexivity (*n* = 7) and/or potential recall bias if interviews were conducted a long time after PP onset (*n* = 10). One study was graded D as it used non‐recorded interviews (Vanderkruik et al. 2024) and there were concerns over the overall dependability and confirmability of findings. Table 3 demonstrates a summary of qualitative appraisal undertaken.

**TABLE 3:** Summary of quality appraisal adapted from Downe et al. (2009).

Author, year	Aims clear?	Participants appropriate for question?	Design appropriate for aims and theoretical perspective?	Methods appropriate for design?	Sample size & sampling justified?	Does the data analysis fit with the chosen methodology?	Reflexivity present?	Study ethical?	Do the data presented justify the findings?	Is the context described sufficiently?	Is there sufficient evidence of rigour?	Score
Engqvist and Nilsson (2014)	Y	Y	Y	Y	UC	Y	Y	Y	Y	N	UC	B
Forde et al. (2019)	Y	Y/UC	Y	Y	Y	Y	Y	Y	Y	Y	Y	A—
Glover et al. (2014)	Y	Y	Y	Y	UC	Y	N	Y	Y	Y	Y	B+
Heron et al. (2012)	Y	Y/UC	Y	Y	Y	Y	N	Y	Y	Y	Y	B
Jefferies et al. (2021)	Y	Y/UC	Y	Y	UC	Y	Y	Y	Y	UC	Y	B
Mcgrath et al. (2013)	Y	Y	Y	Y	UC	Y	Y	Y	Y	Y	Y	A—
Roberts et al. (2018)	Y	Y	Y	Y	Y	Y	Y	Y	Y	UC	UC	B
Robertson and Lyons (2003)	Y	Y	Y	Y	Y	Y	N	Y	Y	Y	UC	B—
Vanderkruik et al. (2024)	Y	UC	UC	N	Y	Y	N	UC	UC	UC	UC	D
Roxburgh et al. (2023)	Y	Y	Y	Y/UC	UC	Y	UC	Y	Y	Y	Y	B
Doucet et al. (2012)	Y	Y	Y	Y	UC	Y	N	Y	Y/UC	Y	UC	B—
Engqvist et al. (2011)	Y	UC	Y	Y	Y	Y	Y	Y	Y	UC	Y	B
Posmontier and Fisher (2014)	Y	Y	UC	Y	Y	Y	Y	Y	Y	Y	Y	B
Wass et al. (2024)	Y	Y	Y	Y	Y	Y	UC	Y	Y	Y	Y	A—
Wyatt et al. (2015)	Y	Y	Y	UC	N	Y	Y	Y	Y	UC	UC	B

## 4 Synthesis Findings

Initially 72 codes were developed and grouped into 10 descriptive labels. Further analysis led to the synthesis of 3 main themes and 9 subthemes. The main themes were: ‘Navigating the unknown’, ‘The double‐edged sword of care’ and ‘Seeking consolation and recovery’. A summary of themes, subthemes and the individual studies they relate to is included in Table 4.

**TABLE 4:** Summary of themes mapped to primary studies.

Themes	Navigating the unknown		The double‐edged sword of care					Seeking consolation and recovery	
Subthemes	Misunderstanding of PP	Seeking accurate and ‘sanitised’ information	Clarity in diagnosis	Stigmatised and judged	Trauma in care	The ‘right’ care for recovery	Tensions in medication use	A need for psychological support	Hope in peer support
Roberts et al. (2018)	x			x					x
Robertson and Lyons (2003)	x	x			x		x		x
Roxburgh et al. (2022)	x	x	X	x	x	x	x		x
Jefferies et al. (2021)	x								x
Wyatt et al. (2015)					x			x	
Forde et al. (2019)	x	x	X	x	x	x		x	x
Mcgrath et al. (2013)	x	x	X	x	x				x
Glover et al. (2014)	x		X	x					
Posmontier and Fisher (2014)	x				x		x	x	
Wass et al. (2024)	x				x	x			
Doucet et al. (2012)	x	x			x			x	
Vanderkruik et al. (2024)	x			x	x		x	x	
Engqvist and Nilsson (2014)					x			x	
Engqvist et al. (2011)		x			x		x		
Heron et al. (2012)	x	x		x	x	x	x	x	x

### 4.1 Navigating the Unknown

This theme discusses women's experiences of navigating an illness that they felt was not well known *(Misunderstanding of PP)*, and women's information needs *(seeking accurate and sanitised information)*.

#### 4.1.1 Misunderstanding of PP

In 13 studies, women reported how they felt there was a lack of knowledge and awareness around PP (Jefferies et al. 2021; Roxburgh et al. 2023; Roberts et al. 2018; Robertson and Lyons 2003; Wass et al. 2024; Forde et al. 2019; Mcgrath et al. 2013; Glover et al. 2014; Posmontier and Fisher 2014; Doucet et al. 2012; Vanderkruik et al. 2024; Heron et al. 2012; Engqvist 2011). Women felt this contributed to their initial symptoms being misunderstood and to delays in accessing treatment. Women felt health care professionals failed to ask the appropriate questions to assess their mental health or misinterpreted their symptoms (Jefferies et al. 2021; Roxburgh et al. 2023). Women referred to how early symptoms of PP such as insomnia and changes in behaviour were sometimes missed or misunderstood by healthcare professionals as ‘adjusting to motherhood’ (Jefferies et al. 2021), or misdiagnosed as postnatal depression (Jefferies et al. 2021; Roxburgh et al. 2023; Posmontier and Fisher 2014). Partners or family members sometimes became advocates for women to health care professionals and helping women access support by highlighting concerns (Heron et al. 2011; Forde et al. 2019) as described in the quote below:

I'd met up with the crisis team the next morning, but they said, no she's fine…and then my husband went in afterwards and he was, ‘actually she's not fine, that's not how she is’ (Woman 13, p.8) (Forde et al. 2019).

Problems with recognition and diagnosis of PP contributed to women feeling fearful, confused and dissatisfied with care (Roberts et al. 2018; Glover et al. 2014; Robertson and Lyons 2003; Roxburgh et al. 2023; Jefferies et al. 2021; Wass et al. 2024). In Robertson & Lyon's study undertaken in the UK, one woman with PP stated:

you have no idea what's going on, what's real and what's not, but when the doctors don't appear to know either that's really scary particularly when they're supposed to make you better. (Sarah, p. 9). (Robertson and Lyons 2003).

Women emphasised the need for education of health care professionals and their families in how to recognise PP and effectively provide support (Jefferies et al. 2021; Vanderkruik et al. 2024; Roberts et al. 2018; Heron et al. 2012; Engqvist et al. 2011; Roxburgh et al. 2023; Forde et al. 2019; Posmontier and Fisher 2014; Wass et al. 2024).

#### 4.1.2 Seeking Accurate and ‘Sanitised’ Information

Informational needs were reported in 7 studies (Roxburgh et al. 2023; Robertson and Lyons 2003; Heron et al. 2012; Doucet et al. 2012; Mcgrath et al. 2013; Forde et al. 2019; Engqvist et al. 2011). Women's ability to understand or access information was compromised by symptoms of PP such as memory loss and women not feeling well enough to self‐seek information in the early stages (Mcgrath et al. 2013; Heron et al. 2012). Women desired information giving to be appropriately tailored to their stage of recovery and for explanation of taken for granted terms such as MBU or PP (Heron et al. 2012; Forde et al. 2019). Difficulties in gaining information contributed to women feeling fearful, isolated and unsupported [13,20]. Women wanted information relating to PP, medication use and safety in breastfeeding (Vanderkruik et al. 2024), simple explanations of frequently used terms such ‘mother and baby unit’ or ‘psychosis’ (Heron et al. 2011; Engqvist et al. 2011), future planning to prevent the risk of relapse (Engqvist et al. 2011; Heron et al. 2012; Doucet et al. 2012; Forde et al. 2019) and positive information about recovery (Mcgrath et al. 2013; Heron et al. 2011; Forde et al. 2019). Some women relied on family members to help them fill in informational gaps (Heron et al. 2012; Forde et al. 2019) however in two studies, women expressed a preference for information sourced from health care professionals with specialist knowledge (Heron et al. 2012; Doucet et al. 2012). Women in the study by Heron et al., also emphasised the need for ‘*censored*’ or ‘*sanitised*’ information (Heron et al. 2012). A few of the participants in this study reported that readily available information on the internet sometimes contained negative associations of PP such as divorce, suicide, infanticide that was ‘unhelpful’ during recovery (Heron et al. 2012). Advice from health care professionals was sought by women as a strategy of maintaining recovery [28]. However, in some studies women reported negative experiences of misinformation (Forde et al. 2019; Roxburgh et al. 2023). A participant recalled her experience of misinformation and how this affected her negatively:

She [my perinatal psychiatrist] said that there's a one in five chance of you getting [PP] again, which she should've said one in two. And she also said to me, which I thought was really unhelpful although she was trying to be helpful, was that she's seen lots of people and no one has ever had it again, no one…So when I got it I felt I failed. (Participant.04. p. 247) (Roxburgh et al. 2022).

### 4.2 The Double‐Edged Sword of Care

Women expressed both positive and negative experiences of accessing care. This theme discusses women's experiences of receiving a diagnosis (*Clarity in diagnosis*), MBU care (*The ‘right’ care for recovery*) and medication support (*Tensions in medication support*). Refutational perspectives of how accessing care was associated with stigma (*Stigmatised and judged*), and trauma particularly in respect to general psychiatric units (*Trauma in care*) *were also discussed*.

#### 4.2.1 Clarity in Diagnosis

Diagnosis was discussed in four UK studies (Forde et al. 2019; Mcgrath et al. 2013; Roxburgh et al. 2023; Glover et al. 2014). Women considered that receiving a diagnosis was important for clarity, and for women to feel safe, secure and trusting of the care given, as well as providing reassurance of hope for recovery (Forde et al. 2019; Mcgrath et al. 2013). A woman in McGrath's study undertaken in the UK described her relief in learning that PP is a known and diagnosable condition:

Even though it was this thing you'd not heard of, it was a relief to know…it does exist, other people have had it before me and there are things that can be done (Participant 6. p. 7) (Mcgrath et al. 2013).

#### 4.2.2 Stigmatised and Judged

From a refutational perspective, some women struggled with receiving a diagnosis. In three studies, diagnosis was associated with stigma and a ‘*label’ of psychosis'* (Roxburgh et al. 2023; Mcgrath et al. 2013; Glover et al. 2014). Some of the women in Glover's study felt judged, perceived as ‘*mad*’ and that people looked at them ‘in a different way*’* following their diagnosis (Glover et al. 2014). Women in the study by McGrath et al., also felt professionals treated them according to their diagnosis rather than their individual needs (Mcgrath et al. 2013).

Stigma around perceptions of mental illness and specifically PP was reported in five studies and reportedly affected how women perceived their self‐identity (Vanderkruik et al. 2024; Roberts et al. 2018; Heron et al. 2012; Mcgrath et al. 2013; Forde et al. 2019). In three UK studies women reported a process of women departing from the perception of themselves as ‘*well‐functioning’* (Forde et al. 2019) and *‘successful’* (Heron et al. 2012) to the adoption of a new identity as ‘mentally ill person*’* or ‘*mental patient’* (Heron et al. 2012; Mcgrath et al. 2013). In the UK study by Robert's, women also described how the depth of stigma felt was exacerbated by the postnatal timing of the illness and societal expectations on motherhood:

It's a double whammy. Not only the stigma of being mentally ill, you've got the stigma of being a mentally ill mother, a bad mum. All pregnant women, all new mums think about ‘am I a good mum?’ and it's a really big thing anyway, but then to bring on mental illness as well, it's massively so. (Participant 8. p. 78) (Roberts et al. 2018).

#### 4.2.3 Trauma in Care

In six studies undertaken in the UK or US, being admitted into a mental health facility was described as traumatic and associated with a loss of control and distress (Roxburgh et al. 2023; Forde et al. 2019; Heron et al. 2012; Mcgrath et al. 2013; Posmontier and Fisher 2014; Vanderkruik et al. 2024). In the UK study by Forde et al., some women reported feeling unsupported during the initial contact with services. This was echoed in another UK study by Roxburgh et al. 2022 where women reported being left ‘sat in the waiting room for I don't know, hours…getting worse and worse…I was hallucinating’ (P11) (p. 247). The involvement of emergency services and police at the point of admission was perceived as distressing (Vanderkruik et al. 2024), ‘*embarrassing*’ (Posmontier and Fisher 2014) and ‘*traumatic*’ (Forde et al. 2019).

Care in hospital settings and general psychiatric units was described using emotive terms such as ‘*prison*’, ‘*jail*’, ‘*trapped*’ (Engqvist and Nilsson 2014; Vanderkruik et al. 2024; Roxburgh et al. 2022) and perceived as the ‘*wrong environment*’ (Heron et al. 2012) and inappropriate for women in the perinatal period or for women who have just given birth (Heron et al. 2011; Vanderkruik et al. 2024; Wass et al. 2024; Robertson and Lyons 2003; Roxburgh et al. 2022). Women reported frustration and anger at not having treatment within MBU facilities and wanted care in a specialist unit where they could stay with their baby (Vanderkruik et al. 2024; Doucet et al. 2012; Wass et al. 2024; Roxburgh et al. 2022; Robertson and Lyons 2003). Separation from their babies added to mothers' distress during admission to general psychiatric units. Some women felt that separation further exacerbated their symptoms:

You can't logically figure out where your babies are or what's happened to them. You get screwed up because you can't check, you think did my baby die—did I kill my baby? It's different from the situation of other mental illnesses because there is a baby involved. The mother's state of being is usually dependent on that baby. (p. 240) (Doucet et al. 2012).

In three studies, separation was associated with guilt over lost time with the baby and their partner (Robertson and Lyons 2003; Wyatt et al. 2015; Heron et al. 2011), and fear over how this would influence their child's development (Wyatt et al. 2015; Robertson and Lyons 2003; Vanderkruik et al. 2024). Although from a refutational perspective, separation was described by one woman to have motivated her within her recovery (Wyatt et al. 2015).

Women described problems with the aesthetics and facilities of general psychiatric units and limited facilities for their perinatal needs, including no facilities to support breastfeeding or pumping (Doucet et al. 2012; Vanderkruik et al. 2024; Roxburgh et al. 2022). Women also referred to how a confusing layout or a lack of cleanliness, privacy and outside facilities added to feelings of distress and restriction:

It just felt horrible… I was just really confused about the whole layout of the place… The toilet was horrible, the shower was horrible, the condition of the place was not very nice… post birth you need…clean sanitation… so I was bleeding a lot still…I needed a bath but you don't have baths in your room. (Participant11.p. 248) (Roxburgh et al. 2022).

In two studies based in the US and Canada, women felt increased isolation due to having limited or restricted contact with family (Vanderkruik et al. 2024; Doucet et al. 2012), and some women were left in ‘*isolation rooms’* with minimal contact with health care professionals (Doucet et al. 2012).

#### 4.2.4 The ‘Right’ Care for Recovery

Mother and baby units (MBUs) were discussed in four UK studies (Heron et al. 2012; Roxburgh et al. 2022; Wass et al. 2024; Forde et al. 2019). MBUs were perceived as a more therapeutic environment (in contrast to general psychiatric units) and the ‘*right’* (Heron et al. 2012) environment for healing and recovery (Heron et al. 2012; Roxburgh et al. 2022; Wass et al. 2024; Forde et al. 2019). Women in the UK study by Roxburgh et al. (2022) described MBUs using words such as ‘*beautiful*’, ‘*spa*’, ‘*holiday*’ and ‘*clean*’:

We took some pictures because it's in a garden and whenever we show someone, they're like ‘where's that beautiful park?’ So it's as if it's like not some mental health‐…It was so beautiful…That's why I say it felt like a spa because it felt so‐…it felt quite luxurious (P04). (Roxburgh et al. 2022).

MBU's were also valued as providing practical support for caring for the baby (Roxburgh et al. 2022; Wass et al. 2024). Women reflected on how MBUs allowed them to interact and shared hopeful stories of women overcoming PP through in‐person interactions (Heron et al. 2011; Roxburgh et al. 2022), or through reading MBU story boards (Heron et al. 2011). In two studies, women felt that MBU staff made more effort to involve partners in decision making about their care, as well as providing support for partners and family in comparison to acute settings (Forde et al. 2019; Roxburgh et al. 2023).

#### 4.2.5 Tensions in Medication Use

Six studies reported women's experiences of medication support (Roxburgh et al. 2023; Robertson and Lyons 2003; Posmontier and Fisher 2014; Heron et al. 2012; Engqvist et al. 2011; Vanderkruik et al. 2024). From one perspective, medication use was perceived as helpful for recovery (Vanderkruik et al. 2024; Posmontier and Fisher 2014; Heron et al. 2012; Engqvist et al. 2011). However, from a refutational perspective, medication use was also perceived to negatively impact women's sense of autonomy, self‐efficacy, personal competency and ability to function (Engqvist et al. 2011; Heron et al. 2012). A woman in a Swedish study exploring recovery from PP reported a sense of feeling like herself again after medication discontinuation:

My real improvement started when I finally stopped all the heavy medication. I was like a zombie. And when I stopped my medication I came back and became my old self again. And I remember it so well, it was such a wonderful feeling! (Informant # 13, woman). (Engqvist and Nilsson 2014).

In three studies [34,38,46] women similarly used the word ‘*zombie*’ to describe how medication made them feel. Medication use was negatively perceived as a passive route to recovery by some women (Heron et al. 2012; Engqvist et al. 2011). Women valued input into their care particularly around treatments and felt that there needed to be a compromise between the use of medications and their need to be able to feel like themselves and function well as mothers (Heron et al. 2012; Roxburgh et al. 2023; Engqvist et al. 2011). In three studies, women felt a lack of control over their treatments and felt there needed to be more information and involvement in decision making about their care (including treatment doses or length of treatment) (Robertson and Lyons 2003; Roxburgh et al. 2023; Heron et al. 2012). Some women reportedly refused medication due to never finding the right dose or fears around breastfeeding (Vanderkruik et al. 2024).

### 4.3 Seeking Consolation and Recovery

This theme discussed women's need to talk about PP both formally and informally. This theme was conceptualised in subthemes: ‘A need for psychological support’ and ‘*Hope in peer support*’.

#### 4.3.1 A Need for Psychological Support

In seven studies, formal counselling/therapy that enabled women to talk about their experiences was discussed [29, 31, 32, 34, 39, 41, 48]. But some women reported experiences of frustration with healthcare services due to the lack of psychological support to help them:

CMHT's you know, you don't fit this tick box now, you don't fit that tick box. But you are left with this life that is destroyed… I needed, I needed something, I needed some kind of psychological support. I needed trauma therapy, I needed somebody to understand that these things were REAL TO ME. (Woman 7). (Forde et al. 2019).

Psychological support was preferred in later stages of recovery and when no longer on high doses of anti‐psychotics (Engqvist and Nilsson 2014; Heron et al. 2012). Some women experienced psychological support by counsellors/psychologists/therapists. Therapy was perceived as good for recovery and helpful in rationalising or making sense of traumatic experiences associated with PP (Heron et al. 2012; Wyatt et al. 2015; Posmontier and Fisher 2014; Engqvist and Nilsson 2014; Forde et al. 2019). Some women also found informal opportunities of staff listening to their concerns– *‘not in a labelled way’–* as useful (Forde et al. 2019).

Whilst therapy was often viewed positively, challenges in therapy included the abrupt end to counselling sessions and difficulties in relating to others in group therapy [31]. Women also reported that psychological support should consider parents' practical needs of flexibility and childcare support (Forde et al. 2019). Some women experienced (Wyatt et al. 2015) or wished for the involvement of family members in therapy and suggested this would be helpful for their own recovery (Heron et al. 2012; Forde et al. 2019). In the UK study by Heron, a woman describes how she perceived a need for family involvement in therapy, to help both parties in understanding each other through recovery:

…trust is a big issue there, you know, a trust has been broken. They don't trust you because you have done all these strange things and you don't trust them because you think they will take you back to hospital. It's taken many, many, many months to solve. I feel if there was some system in place, where they could refer you to psychotherapy and the whole family would be involved so they can understand and you can understand them, it would definitely speed up recovery. (Heron et al. 2012).

#### 4.3.2 Hope in Peer Support

The value of women sharing their lived experiences with others diagnosed with PP was reported in seven studies (Roxburgh et al. 2022; Heron et al. 2012; Forde et al. 2019; Roberts et al. 2018; Robertson and Lyons 2003; Jefferies et al. 2021; Mcgrath et al. 2013). This included women engaging with peer support charities (Forde et al. 2019; Heron et al. 2012), in‐person interaction in MBUs (Heron et al. 2011; Roxburgh et al. 2022), MBU story boards (Heron et al. 2011), online social media pages/blogs, attending conferences on PP (Jefferies et al. 2021) and meeting women with shared experiences (Robertson and Lyons 2003; Roxburgh et al. 2022; Mcgrath et al. 2013). In UK and Australian studies, some women reported feeling isolated in their experience of PP and used words such as ‘*freak*’ (Robertson and Lyons 2003; Jefferies et al. 2021), ‘*alien*’ or ‘*monster*’ (Roberts et al. 2018) to describe how they felt. Stigma was a reported barrier to accessing peer support (Forde et al. 2019), disclosure when mixing with groups of mothers (Heron et al. 2012) and help‐seeking behaviours (Vanderkruik et al. 2024) in UK and US studies. Peer support helped to alleviate isolation, helped women challenge internalised stigma around PP (Roxburgh et al. 2022; Heron et al. 2012; Forde et al. 2019; Roberts et al. 2018; Robertson and Lyons 2003; Jefferies et al. 2021). The quotes below from women in Jefferies' and Forde's studies describe how peer support helped women feel less alienated and brought hope for recovery:

…I do like talking about it, because it's a big thing to just keep inside. …our stories are, apart from maybe the treatment, … are all very similar from what I've heard from the others. We've all been through very similar things, and it was really good to know that I wasn't the only one, and not a total freak. (Violet) (Jefferies et al. 2021).

I think it's helped me not to feel like I'm alone because… reading things from umm, action on postpartum psychosis [APP] and talking to the other women on the ward where I was, we all had different things, some people had had psychosis, and knowing I wasn't on my own umm and that I wasn't going mad and it was a real thing and… umm, that we would get better, definitely, definitely helped (Woman 8). (Forde et al. 2019).

#### 4.3.3 Line of Argument

A central narrative in women's stories is the need to be seen and cared for as a mother and not just an illness. Evidence suggests that women can benefit from specialist care and support that recognises their psychiatric needs alongside perinatal and parenting needs. Care in general psychiatric units contributed to stigma, frustration, dissatisfaction and was generally considered inappropriate. Specialist care that was desired includes education for staff and family in supporting women with PP, provision of accurate and sanitised information, being cared for in a MBU and therapeutic use of medication to facilitate improvement of clinical symptoms whilst also allowing the mother to effectively function. Other important considerations include psychological support to help make sense of the experience of PP, and peer support to relieve isolation and to lessen the stigma felt by women. Health care provisions have the potential to be restorative or traumatic to women. A collaborative and person‐centred approach is needed to ensure care and support is attentive to and addresses women's needs.

## 5 Discussion

The purpose of this review was to identify and synthesise qualitative literature that explored women's experiences of care and support following PP. This review reported on women's experiences of early help‐seeking, inpatient admission, medication support, psychological support and peer support. Recommendations to care have been offered and are presented in Table 5 with further discussion below.

**TABLE 5:** Recommendations to care.

Aspect of care	Recommendation	Purpose
Informational support	Education of health care professionals and informal support networks	To support recognition of PP and early help seeking
		To support appropriate and correct information giving about PP
Medication support	Further involvement of women in decision making about treatments	To balance recovery from psychiatric symptoms with side effects women experience
		To enhance women's sense of autonomy and control over their care
Inpatient treatment	MBU care	To support joint admission with baby. MBU care was viewed by mothers as more therapeutic and appropriate than general psychiatric care.
Psychological support	Provide opportunities for formal and informal psychological support.	Provide opportunities for women to process experience of PP and potential trauma.
	Provide formal psychological support in later stages of recovery.	Formal psychological support was preferred by women in later stages of recovery when no longer on high doses of Anti‐psychotic medications.
	Provide flexibility and practical arrangements around childcare.	To increase accessibility for women to access support.
	Peer support	Peer support may help to relieve isolation for women and gave women hope for recovery.

### 5.1 Early Support Seeking

Issues in early help seeking for women with PP were identified in this review. These included issues in recognition of PP, issues in diagnosis, stigma and lack of awareness or education of support needed. Women with PP reported issues in early support seeking due to misunderstanding of PP symptoms by healthcare professionals and wider family. This finding is consistent with other studies involving women with perinatal mental health conditions whereby women report lacking information around perinatal mental health issues (Ayres et al. 2019) and report negative experiences with healthcare professionals at disclosure (Rice et al. 2022). Women in this review highlighted that issues in PP recognition and support could be supported through education and training of healthcare professionals and informal support networks in understanding PP (Jefferies et al. 2021; Vanderkruik et al. 2024; Roberts et al. 2018; Heron et al. 2012; Engqvist et al. 2011; Roxburgh et al. 2023; Forde et al. 2019).

The stigma of a mental health condition such as PP was found to be a barrier to accessing mental health support during the perinatal period (Sambrook Smith et al. 2019; Mcgrath et al. 2013; Schofield et al. 2023). Mothers who experience perinatal mental health conditions may experience stigma in relation to negative societal views of mental health, and fear judgement from others in relation to their parenting capabilities (Daehn et al. 2022). Stigma may influence women seeking support and cause women to downplay or conceal their symptoms (Schofield et al. 2023). Women in this review found peer support helpful in alleviating stigma from diagnosis. Peer support may be helpful in reducing stigma and beneficial in decreasing isolation, helping women accept the problem and seek further support (Rice et al. 2022).

Difficulties in the recognition of PP may reflect the rare prevalence of the condition and the challenges in the diagnosis of PP. The definition of PP is contentious as there is no universally accepted definition (Sharma et al. 2022). Development of official diagnostic criteria may support the development of official treatment algorithms, prevention plans for subsequent pregnancies, facilitate improvements in screening and identification of PP, and aid prompt detection and treatment. Some women felt diagnosis was helpful in providing clarity; however, it is worth noting that some women found diagnosis as causing de‐personalisation of care and stigmatising. This highlights the importance of ensuring that care is person‐centred rather than disease‐centred care.

### 5.2 In‐Patient Care

As identified within the review some women experienced the process of psychiatric admission traumatic, and stigmatising to women's self‐identity. Women in these studies largely accessed general psychiatric units, with fewer accessing MBUs, and other services. Women cared for in hospital settings were perceived to experience a shift of identity from mother to ‘*mental health patient*’‐ with the latter being associated with stigma, isolation, and a loss of control (Roxburgh et al. 2023; Forde et al. 2019; Heron et al. 2012; Mcgrath et al. 2013; Posmontier and Fisher 2014; Vanderkruik et al. 2024). Separation away from their babies and families caused further trauma for women (Vanderkruik et al. 2024; Doucet et al. 2012; Wass et al. 2024; Roxburgh et al. 2022; Robertson and Lyons 2003; Heron et al. 2012; Wyatt et al. 2015). Challenges in experiencing stigma, managing identity, separation of parents and children following mental illness have been discussed elsewhere (Dunn et al. 2023). The ability to pursue the role of motherhood is of great importance to mothers with perinatal mental health illnesses (Mowbray et al. 2001). There is a need for improved provisions to allow parents to continue in their parenting role whilst receiving psychiatric treatment. Co‐admission for parents with severe mental illness and their children, has shown improved outcomes for both (Dunn et al. 2023). Inpatient care should engage with the parenting identity of the mother as an aspect of recovery and offer support for both (Dunn et al. 2023). This would potentially aid a strength‐based approach to care that is focused on regaining self‐identity and competency, rather than on pathology and deficit. Consistent with other literature, MBUs were perceived to be more specialised to meet women's perinatal needs and were viewed more positively (Griffiths et al. 2019; Howard et al. 2022). MBUs were seen as ‘therapeutic’ and supportive to their psychiatric, perinatal and parenting needs. There is a need for more resources to make MBU care more available and accessible.

### 5.3 Psychological Support

Providing opportunities for psychological support both formally and informally helped women in their recovery and rebuilding of self‐identity (Heron et al. 2012; Roxburgh et al. 2022; Wass et al. 2024; Forde et al. 2019). Further, informal peer support aided connection with others and helped restore women's self‐identity. Women in these studies referred to the need for practical considerations in flexibility and childcare to support women in accessing support. These findings echo those found elsewhere (Daehn et al. 2022), emphasising the importance of addressing practical barriers to enable and encourage access to timely support.

### 5.4 Medication Support

Women in this synthesis highlight factors such as inadequate knowledge about PP and treatments, medication suitability for breastfeeding, and unwanted side‐effects as affecting women's experiences and sometimes decisions to discontinue treatment (Roxburgh et al. 2023; Robertson and Lyons 2003; Posmontier and Fisher 2014; Heron et al. 2012; Engqvist et al. 2011; Vanderkruik et al. 2024). Wider studies have found that the suitability of treatment, women's knowledge, or insight into mental disorders may contribute to medication non‐adherence (Laranjeira et al. 2023; Chakrabarti 2014). Stigma due to taking medications in pregnancy or the postnatal period, as well as lack of professional advice/care planning, may also contribute to non‐adherence to treatment (Kobylski et al. 2024). As non‐adherence is a common reason for readmission to psychiatric units (Haddad et al. 2014), this is an area where more work is needed. Most of the current research focuses on adherence/non‐adherence in respect to psychiatric illness in the general population and less on specifically mothers with psychiatric illnesses (Deng et al. 2022). Further research exploring treatment adherence during pregnancy and the postpartum period is needed to understand how factors within the perinatal period influence women's decisions to adhere or not adhere to treatments and to better tailor treatments to their needs.

### 5.5 Strengths and Limitations

The strengths of this review include the use of a systematic approach, involvement of multiple reviewers, and use of the eMERGe reporting guidance. This review generated findings based on 15 qualitative studies in high‐income countries and included 229 women's experiences of care from a variety of care settings. To our knowledge, the review included all available primary studies on this topic. Data analysis was discussed within the review team, and this helped to alleviate researcher bias. Data analysis was conducted with the highest scoring papers first, finishing with the lowest scoring papers, aiding credibility in the findings presented. The majority of findings were based on women's experiences within in‐patient general psychiatric units, with minimal research on MBUs and experiences after discharge. Participants in these studies reported experiences of PP from 2 months to 23 years. It could be suggested that some studies may be adversely impacted by recall bias. Additionally, participants from these studies were largely white, well‐educated, or had a partner; therefore, it is not clear how well these findings would transfer to women with different racial backgrounds, decreased social support, low socio‐economic status, or from low‐income backgrounds. These gaps highlight a need for further research with women from these demographics. Strong social support has been indicated as supportive for early help‐seeking for women in other studies (Daehn et al. 2022) and access to support needs to be considered for women who may have low social support.

## 6 Conclusion

The qualitative systematic review found that the majority of women experienced care in general psychiatric settings, with few women accessing MBU settings. General psychiatric settings were viewed as inappropriate, and specialised care that considers women's needs as mothers was preferred. Future care improvements should consider the appropriateness of the clinical environment for mothers, including provisions for mothers to stay with their infants. There were additional issues found in respect to medication use, and future research and health care practitioners should consider the niche factors affecting treatment adherence in women with PP. Further education and training of health care professionals to support women with PP may aid in this and in other areas, including recognition of PP and further support in information giving. Psychiatric admission can be a traumatic and stigmatising experience that may conflict with women's self‐identity. Women with PP may also benefit from psychological support to address psychological and emotional trauma that may be associated with admission. Peer support may also alleviate issues around stigma, provide women opportunities to talk about their experience of PP, and provide reassurance for recovery.

## Author Contributions

V.C., G.T., V.M., G.S. made substantial contributions to conception and design, or acquisition of data, or analysis and interpretation of data; V.C., G.T., V.M. involved in drafting the manuscript or revising it critically for important intellectual content; V.C., G.T., V.M., G.S. given final approval of the version to be published. Each author should have participated sufficiently in the work to take public responsibility for appropriate portions of the content; V.C., G.T., V.M. agreed to be accountable for all aspects of the work in ensuring that questions related to the accuracy or integrity of any part of the work are appropriately investigated and resolved.

## Ethics Statement

The authors have nothing to report.

## Consent

The authors have nothing to report. All studies included within the review had participant consent and ethics approval.

## Conflicts of Interest

The authors declare no conflicts of interest.

## Data Availability

All data generated or analysed during this study are included in this published article and is available via the published articles that are referenced in this review.
